# Stimulation of the PD-1 Pathway Decreases Atherosclerotic Lesion Development in Ldlr Deficient Mice

**DOI:** 10.3389/fcvm.2021.740531

**Published:** 2021-11-01

**Authors:** Hendrika W. Grievink, Virginia Smit, Robin A. F. Verwilligen, Mireia N. A. Bernabé Kleijn, Diede Smeets, Christoph J. Binder, Hideo Yagita, Matthijs Moerland, Johan Kuiper, Ilze Bot, Amanda C. Foks

**Affiliations:** ^1^Division of BioTherapeutics, Leiden Academic Centre for Drug Research (LACDR), Leiden University, Leiden, Netherlands; ^2^Centre for Human Drug Research, Leiden, Netherlands; ^3^Department of Laboratory Medicine, Medical University of Vienna, Vienna, Austria; ^4^Department of Immunology, Juntendo University, Tokyo, Japan; ^5^Department of Clinical Pharmacy and Toxicology, Leiden University Medical Center, Leiden, Netherlands

**Keywords:** atherosclerosis, immunology, T cells, coinhibitory pathways, immunotherapy

## Abstract

**Aim:** Signaling through the coinhibitory programmed death (PD)-1/PD-L1 pathway regulates T cell responses and can inhibit ongoing immune responses. Inflammation is a key process in the development of atherosclerosis, the underlying cause for the majority of cardiovascular diseases. Dampening the excessive immune response that occurs during atherosclerosis progression by promoting PD-1/PD-L1 signaling may have a high therapeutic potential to limit disease burden. In this study we therefore aimed to assess whether an agonistic PD-1 antibody can diminish atherosclerosis development.

**Methods and Results:** Ldlr^−/−^ mice were fed a western-type diet (WTD) while receiving 100 μg of an agonistic PD-1 antibody or control vehicle twice a week. Stimulation of the PD-1 pathway delayed the WTD-induced monocyte increase in the circulation up to 3 weeks and reduced T cell activation and proliferation. CD4^+^ T cell numbers in the atherosclerotic plaque were reduced upon PD-1 treatment. More specifically, we observed a 23% decrease in atherogenic IFNγ-producing splenic CD4^+^ T cells and a 20% decrease in cytotoxic CD8^+^ T cells, whereas atheroprotective IL-10 producing CD4^+^ T cells were increased with 47%. Furthermore, we found an increase in regulatory B cells, B1 cells and associated atheroprotective circulating oxLDL-specific IgM levels in agonistic PD-1-treated mice. This dampened immune activation following agonistic PD-1 treatment resulted in reduced atherosclerosis development (*p* < 0.05).

**Conclusions:** Our data show that stimulation of the coinhibitory PD-1 pathway inhibits atherosclerosis development by modulation of T- and B cell responses. These data support stimulation of coinhibitory pathways as a potential therapeutic strategy to combat atherosclerosis.

## Introduction

Atherosclerosis is a chronic autoimmune disease characterized by the accumulation of lipids and immune cells, such as macrophages and pro-atherogenic IFNγ-producing Th1 cells, in the atherosclerotic plaque (1, 2). During disease progression, immune cells respond to atherosclerosis-specific antigens, such as apoB100, the primary protein in low-density lipoprotein (LDL), which are presented via MHC molecules on the surface of antigen-presenting cells (APCs). Subsequent activation of immune cells is regulated by a network of costimulatory and coinhibitory molecules present on both T cells and APCs. The most familiar costimulatory network is the B7/CD28 family, which has proven to be detrimental for Th1-driven atherosclerosis (3). In the past two decades, interference in other costimulatory networks, including the CD40-CD40L and OX40-OX40L pathways, confirmed its potential to inhibit experimental atherosclerosis and to target a broad range of immune responses involved in this disease process (4, 5). Whereas costimulatory molecules need to be suppressed to dampen the overactive immune system in atherosclerosis, signaling through coinhibitory pathways must be promoted. The interaction of programmed death (PD)-1 with PD-L1/2 forms such a coinhibitory pathway, and can inhibit proliferation, cytokine production, cytolytic function, and survival of T cells (6). PD-1 expression is not restricted to activated T cells (7, 8), but can also be upregulated on B cells and certain dendritic cells upon antigen stimulation (7, 9). PD-L1 is expressed by a large variety of cell types, including T cells, macrophages, dendritic cells and endothelial cells (10–12). Previously it has been shown that the PD-1/PD-L1 pathway is a key regulator of many autoimmune diseases, including rheumatoid arthritis (13), multiple sclerosis (14), and cardiac inflammation (15).

In cardiovascular disease patients, altered levels of circulating PD-1 and PD-L1 expressing cells have been reported (16, 17). For example, PD-1 expression on circulating T cells was decreased compared to healthy control individuals and concomitantly, decreased PD-L1 expression on APCs was reported, which corresponded to increased T cell responses (18). Recent proteomics analysis of human atherosclerotic plaques revealed the presence of PD-1 expressing T cell populations inside the advanced atherosclerotic plaque as well (19). Moreover, PD-1/PD-L1 deficiency aggravates experimental atherosclerosis in LDL receptor deficient (Ldlr^−/−^) mice (20–22), with increased numbers of pro-atherogenic CD4^+^ and CD8^+^ T cells in the plaque. It is however unknown whether stimulation of PD-1 signaling can inhibit atherosclerosis. Previous studies have shown that stimulation of coinhibitory molecules, such as CTLA-4 and BTLA, suppressed pro-atherogenic T- and B cell immunity and decreased atherosclerosis development in ApoE^−/−^ and Ldlr^−/−^ mice, respectively (23, 24). In addition, Seko et al. showed that treatment of C3H/He mice with an agonistic PD-1 antibody protected against virus-induced myocarditis (25).

Together, these findings fuel the hypothesis that stimulation of the PD-1 pathway can limit the overactive immune system during atherosclerosis development and thus inhibit plaque progression. Therefore, Ldlr^−/−^ mice fed a western-type diet (WTD) were treated with a stimulatory PD-1 antibody for either 2 or 6 weeks to determine the effects on atherosclerosis development and the atherosclerosis-related immune response.

## Methods

### Animals

Ldlr^−/−^ mice on a C57Bl/6J background were purchased from Jackson Laboratory (Sacramento, CA, USA) and bred in-house. Animals were kept under standard laboratory conditions; food and water were provided *ad libitum*. In order to develop atherosclerotic lesions, female mice (8–12 weeks old) were fed a western-type diet [WTD, 0.25% cholesterol, 15% cocoa butter (SDS, Essex, UK)] for 2 or 6 weeks. The agonistic PD-1 antibody (25) (clone: PIM-2, 100 μg/mouse), isotype control (Tebu-Bio, Heerhugowaard, The Netherlands) or PBS were injected intravenously twice a week in 100 μl volumes. Mice were randomized over the groups using baseline age, weight and cholesterol levels. During the experiments, blood samples were obtained by tail vein bleeding. At the end of experiments, mice were anesthetized by a subcutaneous injection of a cocktail containing ketamine (40 mg/ml), atropine (0.1 mg/ml), and xylazine (8 mg/ml). Mice were bled followed by perfusion with phosphate-buffered saline (PBS) through the left cardiac ventricle. Total white blood cell count and monocyte content in blood were analyzed using an automated XT-2000iV veterinary hematology analyzer (Sysmex Europe GMBH, Norderstedt, Germany). All animal work was performed in compliance with the Dutch government guidelines and the Directive 2010/63/EU of the European Parliament. Experiments were approved by the Ethics Committee for Animal Experiments of Leiden University.

### Cell Preparation

Upon sacrifice, K_2_EDTA anti-coagulated blood, serum, spleens and hearts were harvested. Single-cell suspensions of spleens were obtained using a 70-μm cell strainer (Greiner Bio-one, Kremsmunster, Austria). WBCs were obtained by lysing the blood and splenocytes with ACK lysis buffer (0.15 M NH_4_Cl, 1 mM KHCO_3_, 0.1 mM Na_2_EDTA, pH 7.3). Hearts were embedded in OCT compound (Sakura, Tokyo, Japan) and stored at −80°C until further processing.

### Flow Cytometry

Cell suspensions were stained using fluorochrome-labeled antibodies. A complete antibody list is provided in Supplementary Table 1. Intracellular cytokine staining was performed after stimulation with 50 ng/mL phorbol 12-myristate 13-acetate (PMA) and 200 ng/mL ionomycin (both Sigma-Aldrich, Deisenhofen, Germany) for 4 h in the presence of brefeldin A (Thermo Fisher Scientific, Waltham, MA, USA). Samples were fixed and permeabilized using the fixation and permeabilization kit (BD Biosciences) prior to intracellular staining. Flow cytometry analyses were performed on a Cytoflex S (Beckman Coulter, Brea, CA, USA) or MACSQuant 16 analyzer (Miltenyi Biotec, Bergisch Gladbach, Germany) and FlowJo software (Treestar, San Carlos, CA, USA) or Flowlogics software (Inivai, Mentone, Australia).

### Immunohistochemistry

To determine plaque size, 10 μm sections of the aortic root were prepared and collected. Mean plaque size was calculated using 5 sequential sections stained with Oil-Red-O (ORO) and hematoxylin (both Sigma Aldrich). Intraplaque collagen and necrotic core content were quantified after staining with Masson's Trichrome staining kit (Sigma Aldrich) according to the manufacturer's protocol. Corresponding sections on separate slides were stained for monocyte/macrophage content with a MOMA-2 antibody (1:1000, rat IgG2b, Serotec Ltd.) followed by a goat anti-rat IgG-alkaline phosphatase antibody (1:100, Sigma-Aldrich). CD4^+^ and CD8^+^ T cells were stained using CD4 (RM4-5, 1:90, ThermoFisher) and CD8a (Ly-2, 1:100, eBioscience) antibodies, and a secondary rabbit anti-rat IgG antibody (BA-4001, Vector), followed by the Vectastain ABC kit (PK-4000, Vector). Color development was achieved using novaRED peroxidase (Vector laboratories) as enzyme substrate. T cells were scored manually. The relative amount of collagen and that of macrophages in the lesions is expressed as the percentage collagen- or MOMA-2-positive area of the total lesion surface area. Spleens were sectioned at a 10 μm thickness and were stained with hematoxylin & eosin (Sigma Aldrich) for white pulp quantification and with ORO and hematoxylin to analyze lipid content. All morphometric analyses were performed in a blinded fashion on a Leica CTR6000B microscope with mikroCam II 20 mp (Bresser) using Leica QWin software (Leica Imaging Systems, UK) or ImageJ (FIJI).

### Serum Measurements

Total cholesterol levels were assessed in serum using an enzymatic colorimetric assay (Roche/Hitachi, Mannheim, Germany). Precipath (Roche/Hitachi) was used as internal standard. Total serum levels of IgM and oxLDL-specific IgM were determined by ELISA as described previously (26).

### Proliferation Assay

Splenocytes were cultured in triplicate in a 96-wells round-bottom plate (2 × 10^5^ cells/well) in RPMI1640 + 10% FCS + 100 U/ml streptomycin/penicillin. Cells were stimulated with anti-CD3 and anti-CD28 (2 μg/ml) for 48 h. Proliferation was measured by addition of 3H-thymidine (0.5 μCi/well, Amersham Biosciences, the Netherlands) for the last 16 h. The amount of 3H-thymidine incorporation was measured using a liquid scintillation analyzer (Tri-Carb 2900R, Perkin Elmer). Responses are expressed as stimulation index (SI): ratio of mean counts per minute of triplicate cultures with stimulation to triplicate cultures without stimulation.

### Statistical Analysis

Data are reported as mean ± standard error of mean (SEM). Differences between groups were calculated using a Student's *t*-test, or one way ANOVA with Dunnet's *post-hoc* analysis when 3 groups were compared. When 3 groups were compared, the control chow group and PD-1 agonist group receiving WTD were both compared to the control WTD group. Statistical analyses were performed using Graphpad Prism version 8 (Graphpad, San Diego, CA, USA). One mouse in the control group did not develop any atherosclerosis and was therefore excluded as an outlier (ROUT method) from the data.

## Results

### PD-1 Stimulation Promotes Anti-atherogenic Immunity

**Figure 1:**
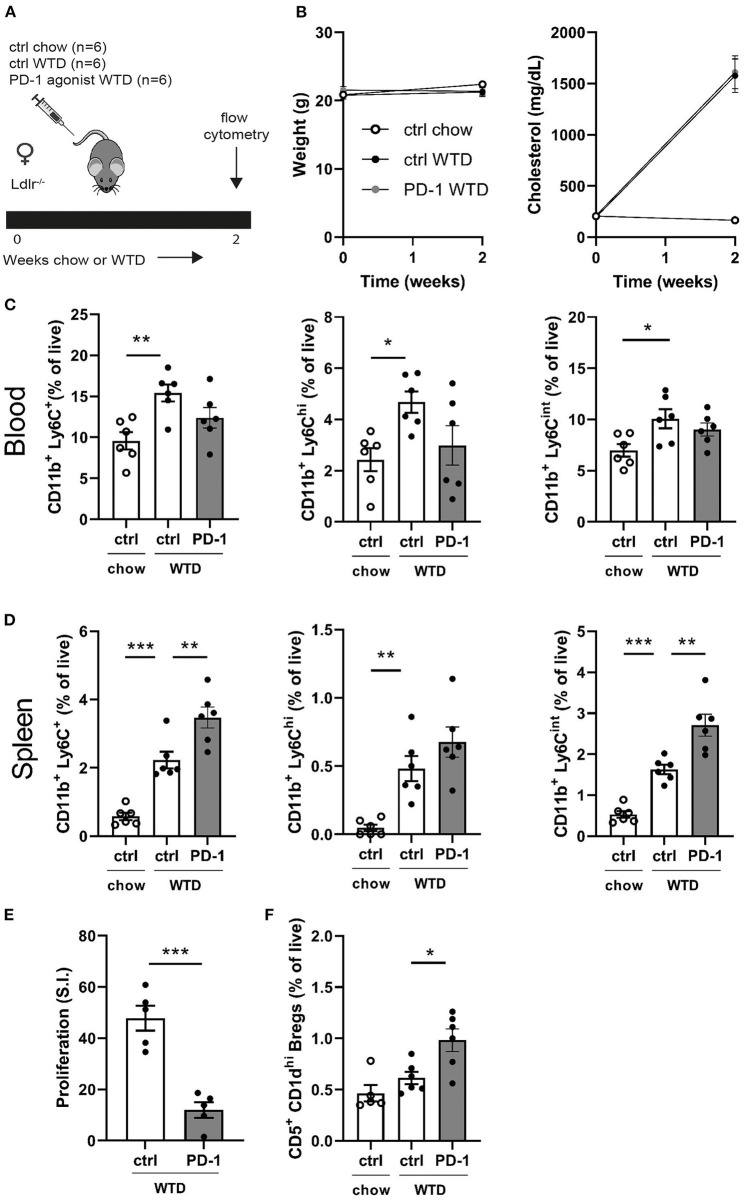
Short term PD-1 stimulation alters monocyte levels and inhibits T cell proliferation. **(A)** Experimental setup. Ldlr^−/−^ mice were fed a WTD or chow diet for 2 weeks while receiving an agonistic PD-1 antibody or control vehicle. **(B)** The weight and serum cholesterol levels were assessed before and after treatment. **(C)** Peripheral blood and **(D)** splenic monocyte percentages were measured by flow cytometry. **(E)** Proliferation of splenocytes after 3 days of stimulation with anti-CD3 and anti-CD28 measured by [3H]thymidine labeling (*n* = 5/group). **(F)** Regulatory B cells in peripheral blood were measured by flow cytometry. Data are displayed as mean ± SEM. Statistics was performed using one-way ANOVA, with *post-hoc* comparison using Dunnett's multiple comparisons test, comparing control chow and PD-1 WTD groups to the control WTD group. *P* ≤ 0.05 are considered significant. *p ≤ 0.05, **p ≤ 0.01, and ***p ≤ 0.001.

To assess the short-term effects of PD-1 stimulation on the immune system, Ldlr^−/−^ mice were treated for 2 weeks with a PD-1 agonist or control vehicle while receiving a WTD diet. Additionally, a control group was kept on chow diet to be able the assess the direct effects of the high cholesterol diet on the immune system (Figure 1A). As shown in Figure 1B, cholesterol levels increased upon WTD administration and no differences in body weight were observed between the experimental groups. Administration of a WTD significantly increased the relative amount of monocytes in peripheral blood (chow: 9.6 ± 1.1% vs. WTD: 15.4 ± 1%, *p* = 0.004, Figure 1C), which was less pronounced in the PD-1 stimulated WTD-fed mice (12.4 ± 1.3%). Similar patterns were seen for both the patrolling (Ly6C^int^) and inflammatory monocyte (Ly6C^hi^) subsets, suggesting that agonistic PD-1 treatment did not affect a specific monocyte subset. Absolute total monocyte numbers as measured by automated hematology analysis revealed a similar trend, albeit these data did not reach significance (*P* = 0.15, Supplementary Figure 1). An additional experiment shows that the relative monocyte effect is PD-1 specific, since monocyte levels upon isotype control treatment (Supplementary Figure 2A) correspond to those of the PBS treated mice (Figure 1C). In contrast, the percentage of splenic monocytes was increased in PD-1 stimulated mice (3.5 ± 0.3%) compared to control WTD (2.2 ± 0.2%, *p* = 0.004) and control chow (0.6 ± 0.1%, Figure 1D). Despite enlarged spleens in mice treated with the PD-1 agonist (Supplementary Figure 3A), we did not observe significant differences in T- or B cell percentages compared to the control vehicle treated mice (Supplementary Figure 3B). As the PD-1/PD-L1 pathway inhibits proliferation of activated T cells (6), the proliferative capacity of splenocytes isolated from either control or PD-1 stimulated mice was measured after stimulation with anti-CD3/CD28 antibodies. As shown in Figure 1E, PD-1 stimulation resulted in a 75% decrease in T cell proliferation compared to the controls (PD-1: 12.0 ± 6.8 S.I. vs. control: 47.8 ± 4.9 S.I., *p* = 0.0003). PD-1 stimulation did not alter the percentages of CD4^+^, CD8^+^ or total CD19^+^ in the periphery (Supplementary Figure 3C). Interestingly, we did observe increased circulating regulatory B cells (Bregs), defined as CD19^+^CD5^+^CD1d^hi^ cells, upon 2 weeks of PD-1 stimulation (PD-1: 1.0 ± 0.1% vs. control WTD: 0.6 ± 0.1%, *p* = 0.02) (Figure 1F).

### Reduced T Cell Activation and Pro-atherogenic IFNγ-Producing T Cells Upon Agonistic PD-1 Treatment

**Figure 2:**
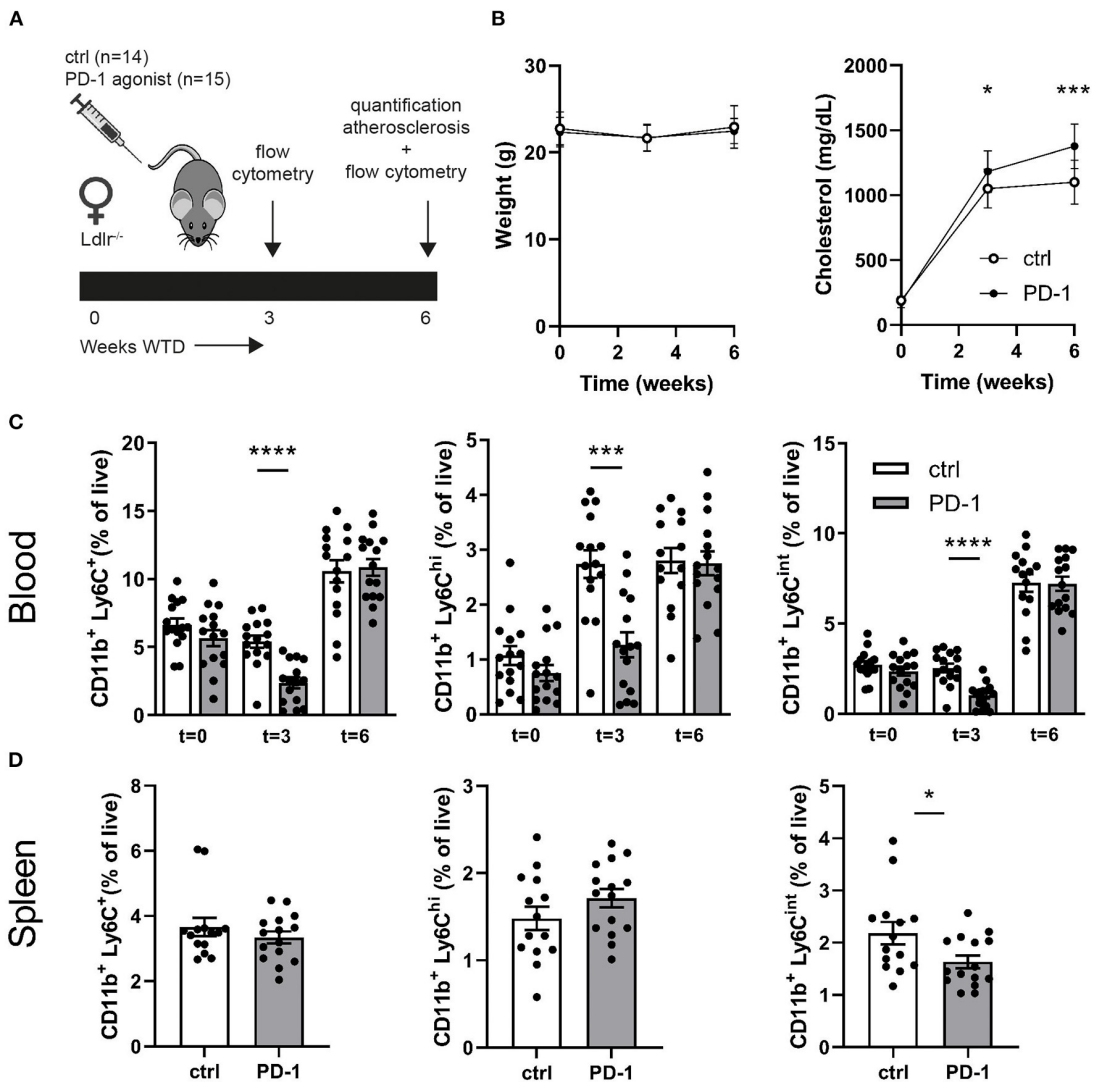
Agonistic PD-1 treatment inhibits WTD-induced increase in circulating monocytes. **(A)** Experimental setup. Ldlr^−/−^ mice were fed a WTD for 6 weeks while receiving an agonistic PD-1 antibody (*n* = 15) or control vehicle (*n* = 14). **(B)** Weight and serum cholesterol levels were assessed before, during and after treatment. Total monocytes (CD11b^+^Ly6G^−^Ly6C^+^), inflammatory monocytes (CD11b^+^Ly6G^−^Ly6C^high^) and patrolling monocytes (CD11b^+^Ly6G^−^Ly6C^int^) were measured by flow cytometry in peripheral blood **(C)** and spleen **(D)**. Mean ± SEM are shown. Statistics was performed using unpaired *T*-test. *P* ≤ 0.05 are considered significant. *p ≤ 0.05, ***p ≤ 0.001, ****p ≤ 0.0001.

**Figure 3:**
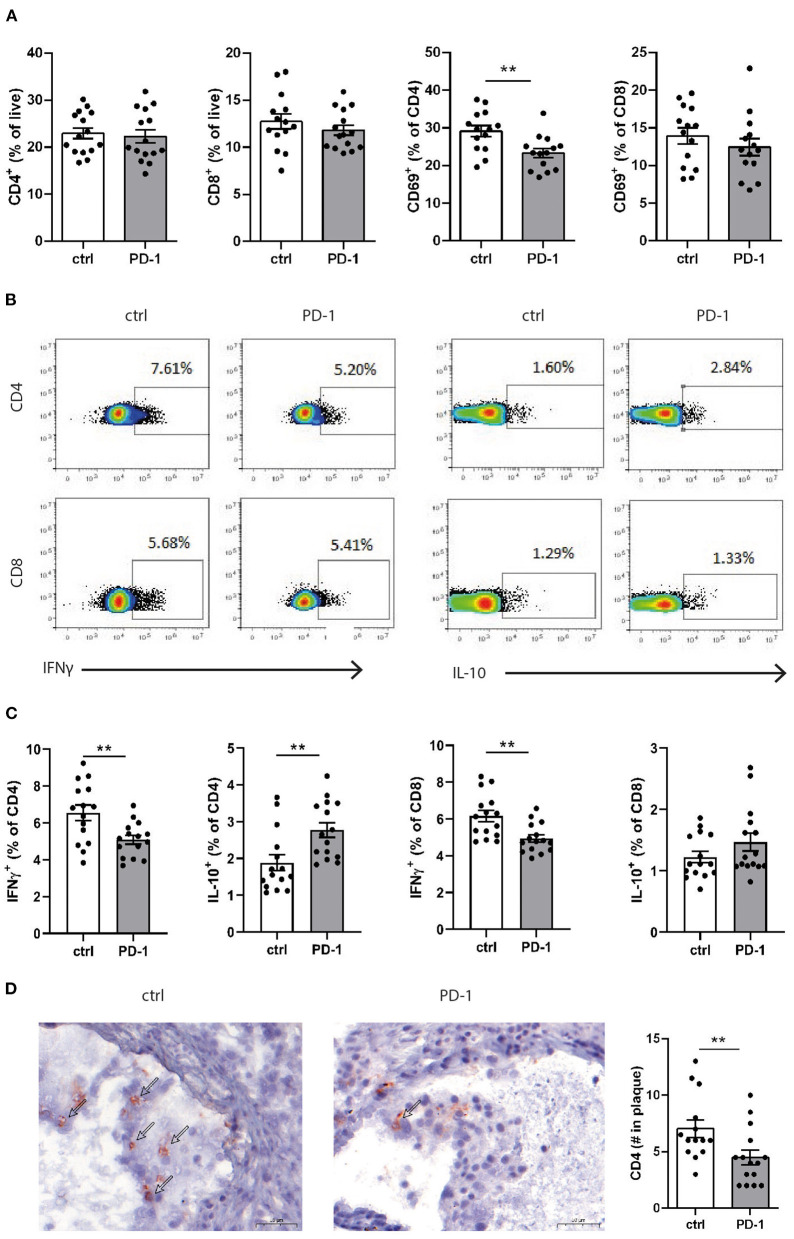
PD-1 stimulation promotes an anti-atherogenic T cell phenotype. **(A)** The amount of CD4^+^ and CD8^+^ T cells and their expression of CD69 was measured in splenocytes by flow cytometry. To measure intracellular cytokines, splenocytes were stimulated for 4 h with PMA/ionomycin and BrefA. **(B)** Representative dotplots and **(C)** data graphs of intracellular IFNγ and IL-10 within CD4^+^ and CD8^+^ T cells are shown. **(D)** CD4^+^ T cell numbers in the atherosclerotic plaque were manually scored. Scale bars indicate 50 μm. Mean ± SEM are shown. Statistics was performed using unpaired *T*-test. *P* ≤ 0.05 are considered significant. **p ≤ 0.01.

Since PD-1 stimulation promoted anti-atherogenic responses in our short-term experiment, we next investigated the immunomodulatory effect of PD-1 stimulation during atherosclerosis development. Ldlr^−/−^ mice were fed a WTD for 6 weeks while receiving an agonistic PD-1 antibody or control vehicle (Figure 2A). During the experiment we observed an increase in serum cholesterol in PD-1 stimulated mice, while no difference in weight was observed between the groups (Figure 2B). In line with our previous findings (Figure 1C), PD-1 stimulated mice show a decrease in circulating monocytes (2.4 ± 0.4%) compared to control mice (5.4 ± 0.5%, *p* = 0.001, Figure 2C) after 3 weeks. At sacrifice, circulating monocyte percentages did not differ anymore between the groups. No differences in relative monocyte content was observed in the spleen after 6 weeks of treatment, except for a significant decrease in patrolling monocytes (PD-1: 1.6 ± 0.1% vs. control: 2.2 ± 0.2%, *p* = 0.03) (Figure 2D). Again, spleen weight was increased in PD-1 stimulated mice, however no differences were found in the relative white pulp content of the spleen (PD-1: 21.6 ± 1.0% vs. control: 23.4 ± 1.0%) and no excess fat depositions were found in spleens of PD-1 agonistic treated mice compared to spleens of control mice (Supplementary Figure 4A). Percentages and absolute values of total CD4^+^ and CD8^+^ T cells were not affected in the spleen (Figure 3A and Supplementary Figure 4B). Similarly, circulating CD4^+^ and CD8^+^ T cells and their activation status was unchanged (Supplementary Figure 5). However, the activation status of splenic T cells, as measured by the expression of activation marker CD69, was decreased in PD-1 agonist treated mice (PD-1: 23.3 ± 4.6% vs. control: 29.2 ± 5.4%, *p* = 0.005) (Figure 3A). Moreover, a significant decrease in pro-atherogenic IFNγ-producing cells was seen in both CD4^+^ and CD8^+^ populations upon PD-1 stimulation (CD4^+^ T cells: PD-1: 5.1 ± 0.9% vs. control: 6.6 ± 1.6% *p* = 0.005; CD8^+^ T cells: PD-1: 4.9 ± 0.8% vs. control: 6.2 ± 1.2% for control, *p* = 0.006) (Figures 3B,C and Supplementary Figure 6), whereas a significant increase in atheroprotective CD4^+^ IL-10-producing cells was found after PD-1 stimulation (PD-1: 2.8 ± 0.8% vs. control: 1.9 ± 0.8%, *p* = 0.003). In the lymph nodes draining from the heart, we did not observe differences in T cell numbers or activation status (data not shown). Locally in the atherosclerotic plaque, a decrease in the number of CD4^+^ T cells after PD-1 stimulation was observed (PD-1: 4.5 ± 0.6 vs. control: 7.0 ± 0.8, *p* = 0.008) (Figure 3D), while the number of CD8^+^ T cells was relatively low and did not significantly differ between the groups (PD-1: 1.2 ± 0.4 vs. control: 0.8 ± 0.2, *p* = 0.61) (Supplementary Figure 5C).

### Increased Circulating B1 Cells and oxLDL-Specific IgM in Agonistic PD-1 Treated Mice

**Figure 4:**
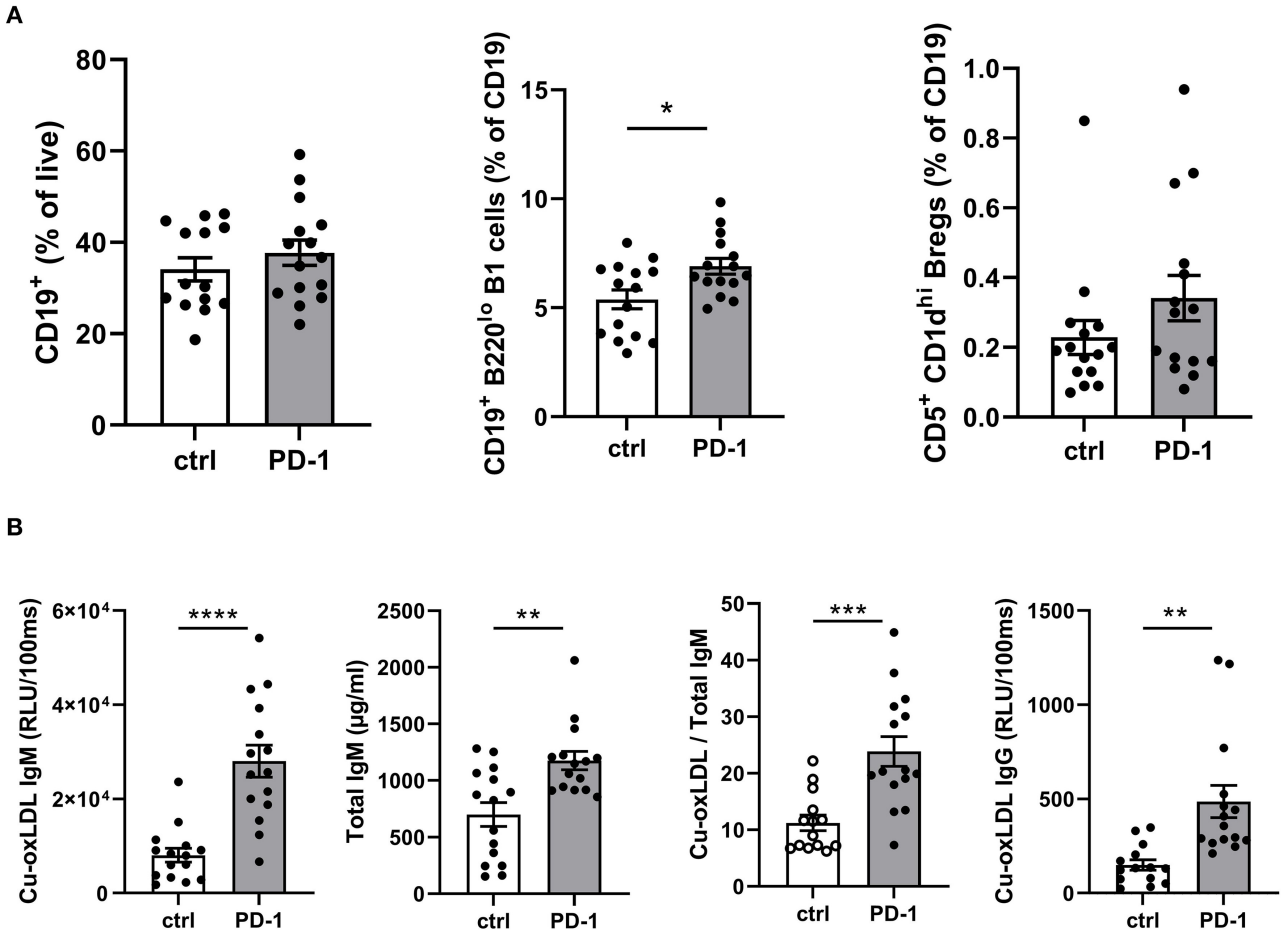
Elevated B1 cells and oxLDL-IgM levels in agonistic PD-1 treated mice. Total CD19^+^ B cells, B1 cells (CD19^+^B220^lo^) and regulatory B cells (CD19^+^CD5^+^CD1d^hi^) were measured by flow cytometry in blood **(A)**. Serum Cu-oxLDL IgM (relative light units (RLU)/100 ms), total IgM (μg/ml), Cu-oxLDL IgM/total IgM ratio, and Cu-oxLDL IgG (RLU/100ms) were measured by ELISA **(B)**. Mean ± SEM are shown. Statistics was performed using unpaired *T*-test. *P* ≤ 0.05 are considered significant. *p ≤ 0.05, **p ≤ 0.01, ***p ≤ 0.001, ****p ≤ 0.0001.

As T cells play an important role in B cell activation, and PD-1 is also expressed on activated B cells, we anticipated PD-1 stimulation may also affect humoral immunity during atherosclerosis development. Although total circulating B cells were unaltered after 6 weeks of PD-1 stimulation, we did observe a significant increase in B1 cells (PD-1: 6.9 ± 1.4% vs. control: 5.4 ± 1.7%, *p* = 0.01, Figure 4A). In line with this finding, total serum IgM levels as well as oxLDL-specific IgM levels were increased by PD-1 stimulation (Figure 4B). Notably, the levels of oxLDL IgM were increased to a greater extent than total IgM, indicating also a relative increase of atheroprotective IgM. Total oxLDL-specific IgG were also increased after PD-1 stimulation. Similarly to the short-term experiment, an increase of circulating Bregs was observed, although not statistically significant (PD-1: 0.34 ± 0.07% vs. control: 0.23 ± 0.05%, Figure 4A).

### PD-1 Stimulation Reduces Plaque Development in the Aortic Root

**Figure 5:**
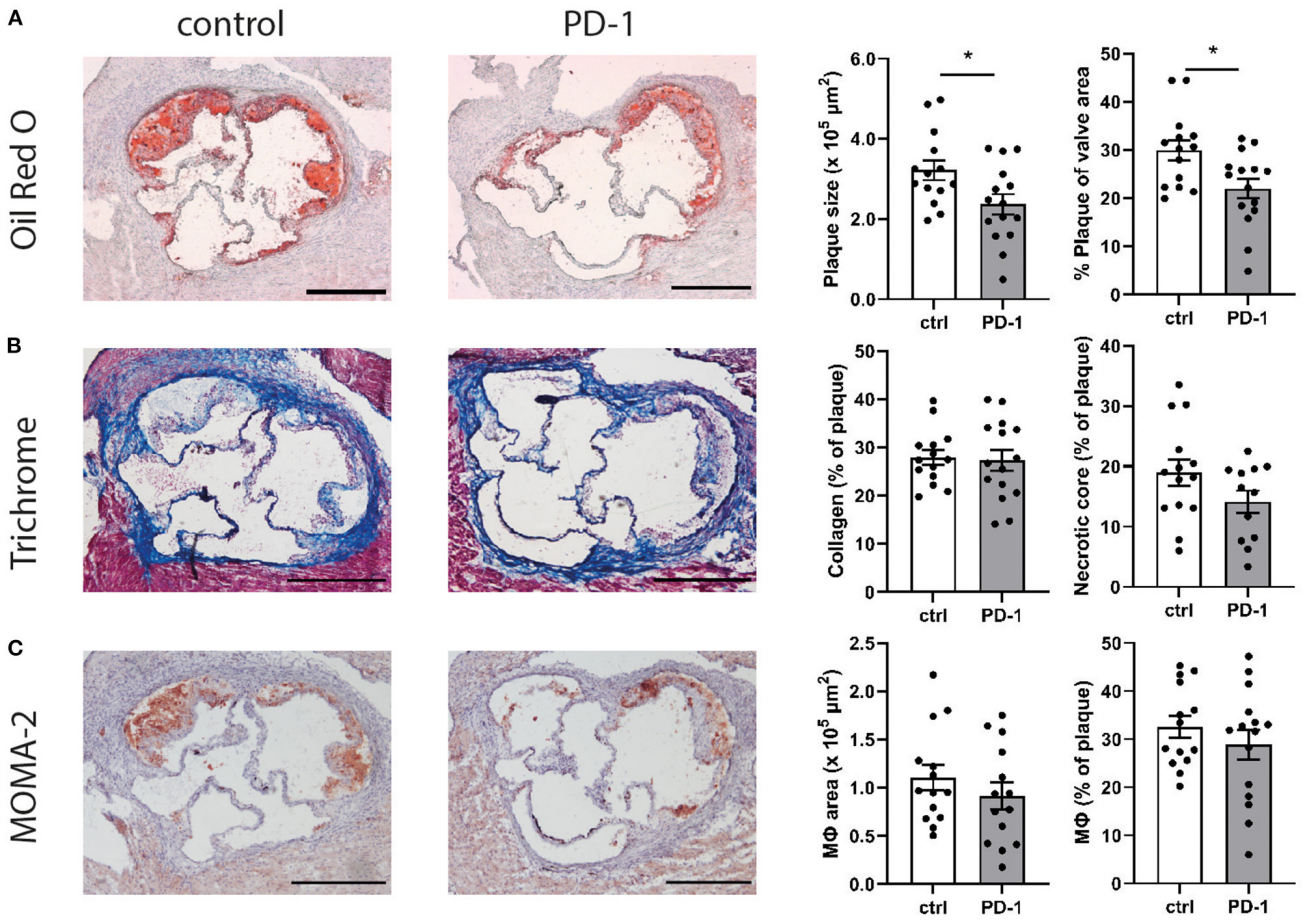
PD-1 stimulation inhibits atherosclerosis development. Aortic root plaque size and composition was determined using an Oil-Red-O staining and representative images of the stainings are shown. Data is shown as total plaque size, and as percentage of total valve area **(A)**. Collagen content and necrotic core size were determined using trichrome staining, and are showed as percentage of the total plaque **(B)**. Macrophage content was determined using MOMA-2 antibody staining, and is shown as total MOMA-2 positive area, as well as the percentage of total plaque **(C)**. Scale bars indicate 250 μm. Mean ± SEM are shown. Statistics was performed using unpaired *T*-test. *P* ≤ 0.05 are considered significant. *p ≤ 0.05.

Given the immunosuppressive effect of PD-1 stimulation, we investigated the effect of PD-1 stimulation on atherosclerotic plaque development. Ldlr^−/−^ mice treated with an agonistic PD-1 antibody showed a 26.4% smaller plaque size compared to the control group (PD-1: 2.40 ± 0.25 × 10^5^ μm^2^ vs. control: 3.22 ± 0.25 × 10^5^ μm^2^, *p* = 0.04) (Figure 5A). Similarly, when plaque size was calculated as a percentage of the total lumen area a significant decrease was seen in PD-1 stimulated mice (PD-1: 23.0 ± 1.8% vs. control: 30.0 ± 2.1%, *p* = 0.02). The percentage of collagen in the plaque was similar in both groups (PD-1: 27.3 ± 2.2% vs. control: 28.0 ± 1.5%, Figure 5B). Likewise, the necrotic core content of the plaque (PD-1: 14.1 ± 1.9% vs. control: 19.0 ± 2.2%) and the macrophage content in the plaque as assessed by MOMA-2 staining (PD-1: 30.5 ± 2.8% vs. control: 32.6 ± 2.3%) did not differ between the groups (Figure 5C).

## Discussion

Immune checkpoint proteins are extremely potent targets to modulate immunity in autoimmune diseases such as cardiovascular disease (23, 24, 27). In this study, we show that stimulation of signaling through the immune checkpoint protein PD-1 inhibits atherosclerotic lesion development in WTD-fed Ldlr^−/−^ mice despite elevated serum cholesterol levels. This is accompanied by a decrease in inflammatory monocytes in peripheral blood in the early stages and a decrease in IFNγ-producing T cells, while atheroprotective IL-10 producing T cells, Bregs, B1 cells and oxLDL IgM levels were increased (20–22).

Previously, it was shown that absence of PD-1/PD-L1/2 signaling can aggravate atherosclerosis by enhancing T cell proliferation, activation of both CD4^+^ as CD8^+^ T cells, and more specifically by increasing pro-atherogenic IFNγ production by T cells (20, 22). In line with these findings, we show that agonistic PD-1 treatment resulted in a strongly impaired proliferative capacity of T cells, with a concomitant shift from Th1-associated IFNγ-producing CD4^+^ cells toward anti-inflammatory IL-10 producing CD4^+^ T cells. This was accompanied by a significant decrease in CD4^+^ T cell numbers in the plaque. This is not surprising, as PD-1 signaling is known to suppress T cell activation and proliferation (28, 29). Reduced IFNγ-producing T cells in our agonistic PD-1 treated mice likely contributed to diminished atherosclerosis, as administration of exogenous IFNγ to ApoE^−/−^ mice resulted in an increase plaque formation (30), while Ldlr^−/−^IFNγ^−/−^ mice developed smaller plaques compared to control (31). In contrast, IL-10 has well-described anti-atherogenic properties (32) and is often associated with regulatory T cells, which can suppress activation and proliferation of immune cells during atherosclerosis, including IFNγ-producing CD4^+^ T cells (33). Despite our observed reduction in T cell proliferation and IFNγ-producing T cells in agonistic PD-1 treated mice, we did not observe a difference in the percentage of Foxp3^+^ CD4^+^ T cells in the circulation (Supplementary Figure 5A). That PD-1 directly acts on pro-atherogenic T cells without affecting Treg levels, was also described by Bu et al. who showed that PD-1 deficiency in Ldlr^−/−^ mice did not alter CD4^+^Foxp3^+^ T cells (21).

Furthermore, we also observed a decrease in IFNγ-producing CD8^+^ T cells, which are considered pro-atherogenic due to their cytotoxic capacity and inflammatory cytokine production (34). This is in line with previous studies in which PD-1^−/−^Ldlr^−/−^ mice showed an increase in pro-inflammatory cytokine expression, including IFNγ by CD8^+^ T cells, and in which PD-1 blockade induced IFNγ production by both CD4^+^ and CD8^+^ T cells (20). Moreover, PD-1 expressing CD8^+^ T cells from patients with atherosclerosis produced more anti-atherogenic IL-10 and less pro-atherogenic cytokines (IFNγ, TNFα) compared to PD-1^−^CD8^+^ T cells, further supporting a protective role for PD-1 in T cell-mediated immunity. Interestingly, CD8^+^ T cells can also control monopoiesis and circulating monocyte levels in atherosclerosis (35), which may have contributed to our observed reduction in circulating monocytes. The latter can also result from reduced T cell activation in PD-1 agonist treated mice as it has been shown that activated T cells can induce pro-inflammatory cytokine secretion by monocytes (36). Lack of this monocyte activation upon PD-1 stimulation possibly prevents influx of new monocytes into the circulation. We show this reduction in monocytes is directly associated with PD-1 stimulation, as treatment with an isotype control resulted in comparable monocyte levels to PBS treatment. Due to limited statistical power in that particular experiment, no concrete conclusion can be drawn on atherosclerosis development. Agonistic PD-1 treatment was only able to delay WTD-induced monocytosis, as relative monocyte levels did not differ between control and PD-1 agonist treated mice after 6 weeks of treatment. Although we have no supportive evidence, the comparable levels of monocytes at sacrifice could be attributed to enhanced monocyte release from the bone marrow in PD-1 agonist treated mice to compensate for the reduced monocytes in the first few weeks of the treatment. As we also observe decreased monocyte levels in the spleen after 3 weeks of treatment, it is also a possibility that monocytes from the splenic reservoir (37) are suppressed during the initial weeks of PD-1 agonist treatment, resulting in reduced circulating monocytes. Notably, we did not observe a difference in macrophage content in the plaque, rendering it unlikely that the observed reduction in monocyte levels during the 1st weeks of the treatment is solely responsible for the observed plaque size reduction upon PD-1 agonism. However, a more detailed analysis regarding macrophage subsets in the plaque may shed more light on the underlying mechanisms involved.

The PD-1/PD-L1/L2 pathway is not only an important negative regulator of T cell responses but can also impact B cell immunity (9, 38). Although PD-L1 expressing B cells have been more extensively investigated, PD-1 is also upregulated on activated B cells and these PD-1^+^ B cells have been linked to CD4 and CD8 T cell suppression previously (9, 38, 39). Within 2 weeks upon PD-1 stimulation, we already observed elevated circulating CD5^+^CD1d^hi^ regulatory B cells, which could possibly exert an atheroprotective role by suppressing T cells. Previously, decreased amount of circulating Bregs were shown in patients with coronary atherosclerosis compared to healthy controls (40) and we showed that adoptive transfer of IL-10^+^ Bregs in Ldlr^−/−^ mice reduced total leukocyte counts, lymphocytes, monocytes and activated T cells in circulation (41). Notably, this adoptive transfer of IL-10^+^ Bregs as well as lack of IL-10 producing B cells in Ldlr^−/−^ mice (42) did not affect plaque size, suggesting our observed increase in Bregs upon PD-1 agonism mainly contributes to reduced inflammation. Finally, we also found elevated levels of B1 cells in the circulation after PD-1 stimulation. B1 cells are considered atheroprotective via their production of primarily IgM natural antibodies directed at amongst others to oxLDL, which can prevent foam cell formation, and facilitate the clearance of apoptotic cells (43). Corresponding to the increase in B1 cells, we observed elevated serum IgM levels and more specifically an absolute and relative increase in oxLDL-specific IgM, which has been shown to inversely relate to the incidence of CVD (44).

Finally, we would like to address a few findings that warrant further research. First of all, our study was performed in female mice. Previous studies investigating the PD-1/PD-L1 pathway were performed in either male (22) or female mice (20), and in both sexes PD-1 or PD-L1 deficiency aggravated atherosclerosis development. Therefore, we do not anticipate sex differences upon PD-1 stimulation in our atherosclerosis model, although further research would be necessary to confirm this. Moreover, we also observed an enlargement of the spleen after PD-1 stimulation, while the amount of splenocytes, white pulp content and fat deposits is similar to those in control mice after 6 weeks of treatment. Previous studies using radioactively labeled monoclonal antibodies show that the spleen is a preferential site of accumulation for therapeutic antibodies (45), which could possibly explain this observation. Finally, despite decreased atherosclerosis, we did observe elevated cholesterol levels in our PD-1 agonist treated mice. This is in contrast to Cochain et al. who found increased cholesterol levels upon PD-1 deficiency in Ldlr^−/−^ mice (22), while cholesterol levels remained unchanged in other studies investigating PD-1/PD-L1 deficiency in atherosclerosis (20, 21). It thus remains to be elucidated whether the observed effect on serum cholesterol levels in our study is directly related to PD-1 agonism or whether this is a secondary effect. Despite the increase in cholesterol levels, we did observe a decrease in atherosclerosis development, suggesting that the atheroprotective effects that we observe on the immune system upon PD-1 treatment are effective in counteracting the increase in serum cholesterol levels. Further research investigating the direct effects on PD-1 agonism on cholesterol metabolism may provide mechanistic insights in this regard.

Given the recent advances in immune modulation as treatment for atherosclerosis (46), our data show that PD-1 stimulation is a possible treatment to reduce atherosclerosis development. Although our study provides novel insights in the role of the PD-1 pathway in atherosclerosis, caution is warranted when using this target for treatment. While dampening the immune system and thus stimulation of the PD-1/PD-L1 axis is a potential pathway to reduce progression of atherosclerosis, activation of the immune system by blockade of the PD-1/PD-L1 pathway has shown promising results in the treatment of several cancers (47). Indeed, a number of patients treated with anti-PD-1 or PD-L1 show cardiotoxicity, mostly myocarditis, especially in combination with anti-CTLA4 treatment (48). Although more research is needed to enhance our knowledge regarding this ambivalence, recently it was reported that melanoma patients receiving anti-PD-1 and anti-CTLA4 treatment did not show a direct effect on atherosclerosis after 10 weeks of treatment (49).

In conclusion, our data show that stimulation of the co-inhibitory PD-1 pathway inhibits atherosclerosis development via modulation of the immune response and supports that stimulation of co-inhibitory molecules can be a potential therapeutic strategy to limit atherosclerosis.

## Data Availability Statement

The raw data supporting the conclusions of this article will be made available by the authors, without undue reservation.

## Ethics Statement

All animal work was performed in compliance with the Dutch government guidelines and the Directive 2010/63/EU of the European Parliament. Experiments were approved by the Ethics Committee for Animal Experiments of Leiden University.

## Author Contributions

HG, JK, IB, and AF designed the research. HG, RV, VS, MB, DS, IB, and AF acquired the data. HG, MB, IB, and AF analyzed the data. HG and AF drafted the manuscript. IB and AF provided critical feedback on the manuscript. All authors provided feedback on the research, analyses, and manuscript.

## Funding

This work was supported by the Dutch Heart Foundation (2018T051 and 2019T107 to AF) associated with the European Research Area Network (ERA-CVD B-eatATHERO consortium). IB was an Established Investigator of the Dutch Heart Foundation (2019T067).

## Conflict of Interest

The authors declare that the research was conducted in the absence of any commercial or financial relationships that could be construed as a potential conflict of interest.

## Publisher's Note

All claims expressed in this article are solely those of the authors and do not necessarily represent those of their affiliated organizations, or those of the publisher, the editors and the reviewers. Any product that may be evaluated in this article, or claim that may be made by its manufacturer, is not guaranteed or endorsed by the publisher.
