# Supplementary material for: Stimulation of the PD-1 Pathway Decreases Atherosclerotic Lesion Development in Ldlr Deficient Mice
Source: Front Cardiovasc Med. 2021 Nov 1;8:740531. doi: 10.3389/fcvm.2021.740531 (PMC8591266; doi:10.3389/fcvm.2021.740531)
Supplement: Supplementary file 1 [file Data_Sheet_1.PDF]

Supplemental data

Stimulation of the PD-1 pathway decreases atherosclerotic lesion development in Ldlr deficient mice

Grievink et al.

Table S1: Flow cytometry antibody list

Marker	Fluorochrome	Clone	Supplier
CD1d	FITC	1B1	Biolegend
CD3e	PE-Cy5	145-2C11	Biolegend
CD4	V500	RM4-5	BD
CD4	PerCP	RM4-5	BD
CD5	PerCP	53-7.3	BD
CD8a	AF700	53-6.7	eBioscience
CD8a	PE-Texas Red	5H10	Invitrogen
CD9	FITC	MZ3	Biolegend
CD11b	PE	M1/70	Biolegend
CD11b	BV605	M1/70	Biolegend
CD11c	FITC	N418	Biolegend
CD19	BV605	6D5	Biolegend
CD21	BV421	7E9	Biolegend
CD23	PE	B3B4	Biolegend
CD43	APC	S11	Biolegend
CD44	PE-Cy7	IM7	Biolegend
CD45	AF700	30-F11	Biolegend
CD62L	APC	MEL-14	eBioscience
CD69	BV510	H1.2F3	Biolegend
CD86	PE-Cy5	GL1	Biolegend
CD93	PE-Cy7	AA4.1	Biolegend
B220	AF700	RA3-6B2	Biolegend
F4/80	BV421	BM8	Biolegend
IgD	APC	11-26c.2a	Biolegend
IgM	FITC	RMM-1	Biolegend
Ly6C	PE-CF594	AL-21	BD
Ly6G	PerCP	1A8	Biolegend
MHC II	eVolve655	M5/114.15.2	eBioscience
**Intracellular marker**			
Foxp3	eFluor 450	FJK-16s	eBioscience
Gata-3	PE	16E10A23	Biolegend
RorγT	BV650	Q31-378	BD
Tbet	PE-Cy7	4B10	eBioscience
IFNγ	AF488	XMG1.2	Biolegend
IL-4	PE-Cy7	11B11	Biolegend
IL-10	APC	JES5-16E3	eBioscience
IL-17A	PE	TC11-18H10.1	Biolegend

PE=Phycoerythrin, BV=Brilliant Violet, AF=Alexa Fluor, FITC=Fluorescein isothiocyanate, APC=allophycocyanin, PerCP=Peridinin-Chlorophyll-protein.

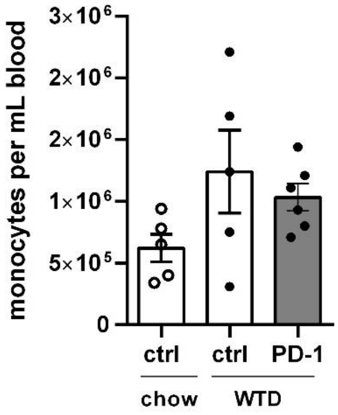

Figure S1: Monocyte levels after 2 weeks of PD-1 stimulation. Ldlr$^{-/-}$ mice were fed a WTD or chow for 2 weeks while receiving a PD-1 agonist or control vehicle. Absolute numbers of monocytes in the circulation were measured using an automated hematology analyser (Sysmex).

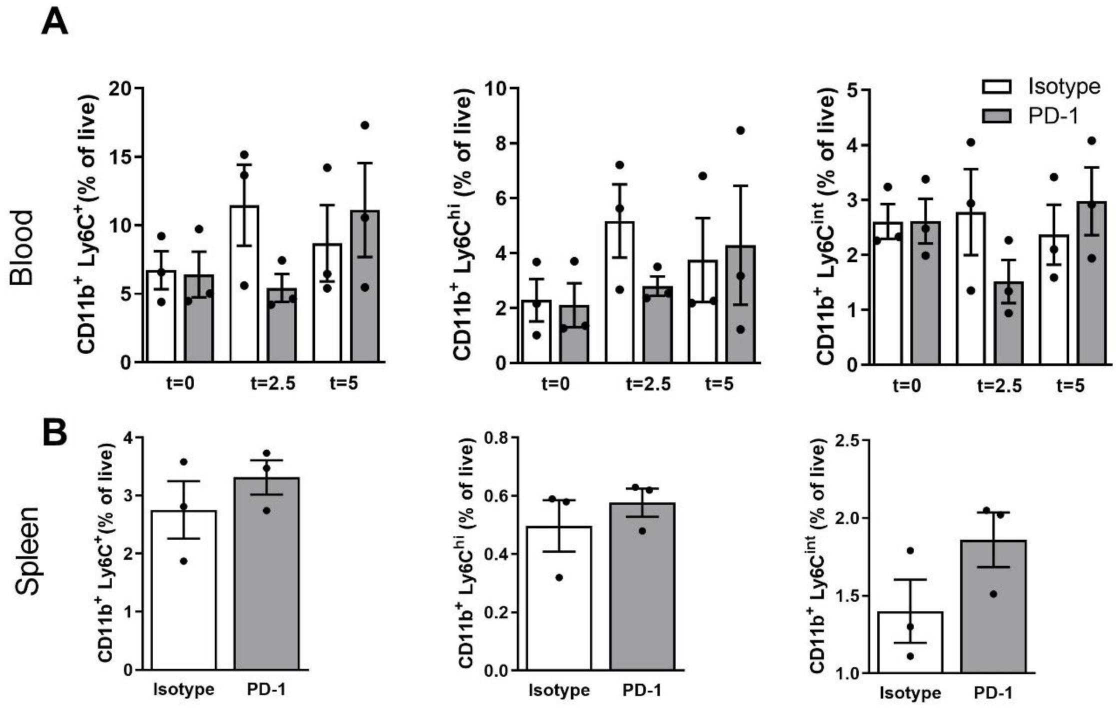

Figure S2: PD-1 specific monocyte decrease after short term PD-1 activation. Ldlr$^{-/-}$ mice were fed a WTD for 5 weeks while receiving a PD-1 agonist or isotype control (n=3/group). The percentage of monocytes was determined with flow cytometry for both blood (A), as well as in the spleen (B). Monocytes were characterized as Ly6C^{+} and Ly6G^{-}. Patrolling monocytes are Ly6C^{int}, inflammatory monocytes Ly6C^{high}. Mean ± SEM are shown.

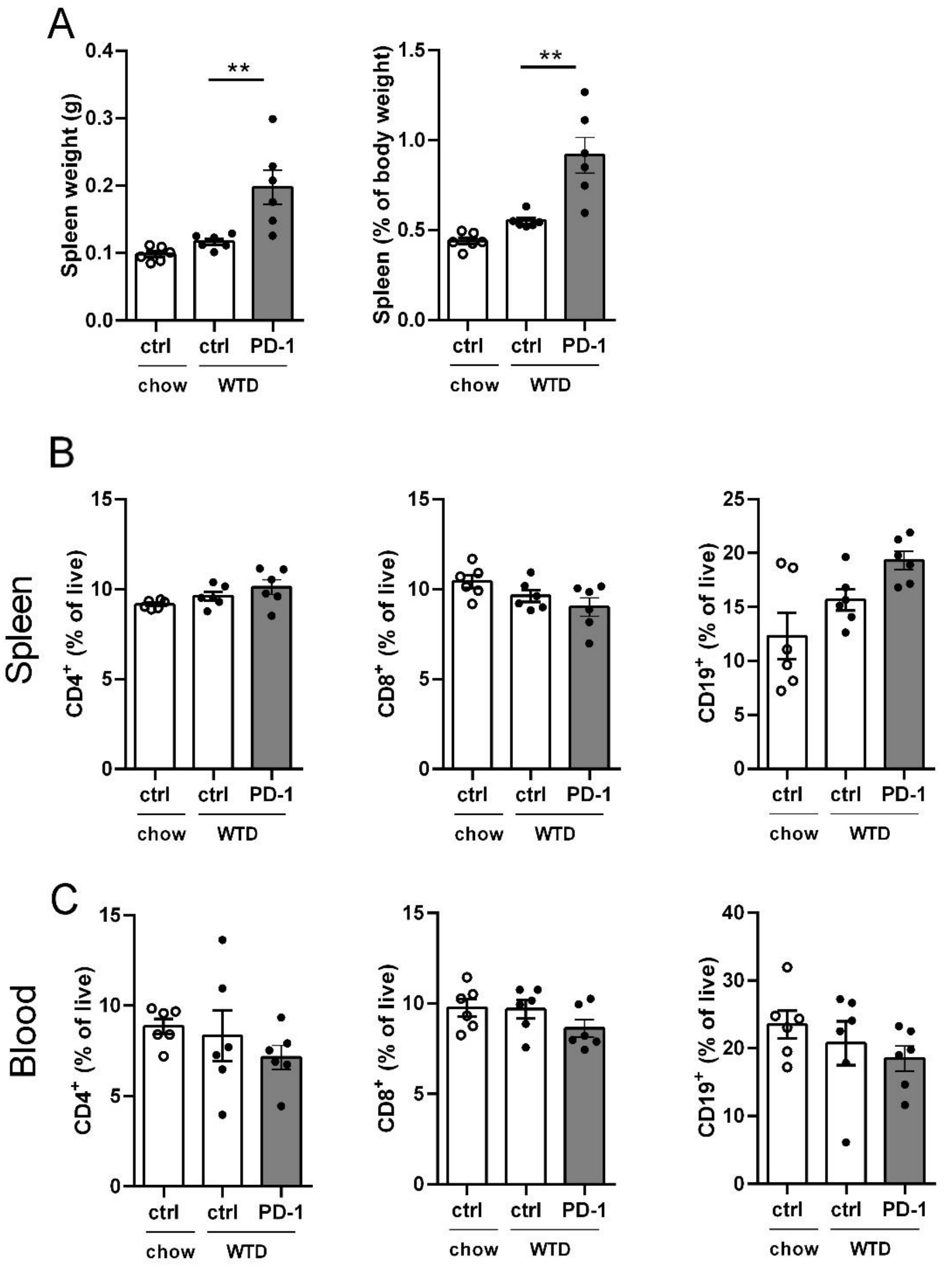

Figure S3: No differences in the percentage of total T and B cells after 2 weeks of PD-1 stimulation. Ldlr$^{-/-}$ mice were fed a WTD or chow for 2 weeks while receiving a PD-1 agonist or control vehicle. Weight of the spleen is shown in grams, and as percentage of body weight (A). The percentage of CD4$^+$, CD8$^+$ and CD19$^+$ lymphocytes was determined with flow cytometry for both the spleen (B), as well as in blood (C). Data are displayed as mean ± SEM. ** $p \leq 0.01$.

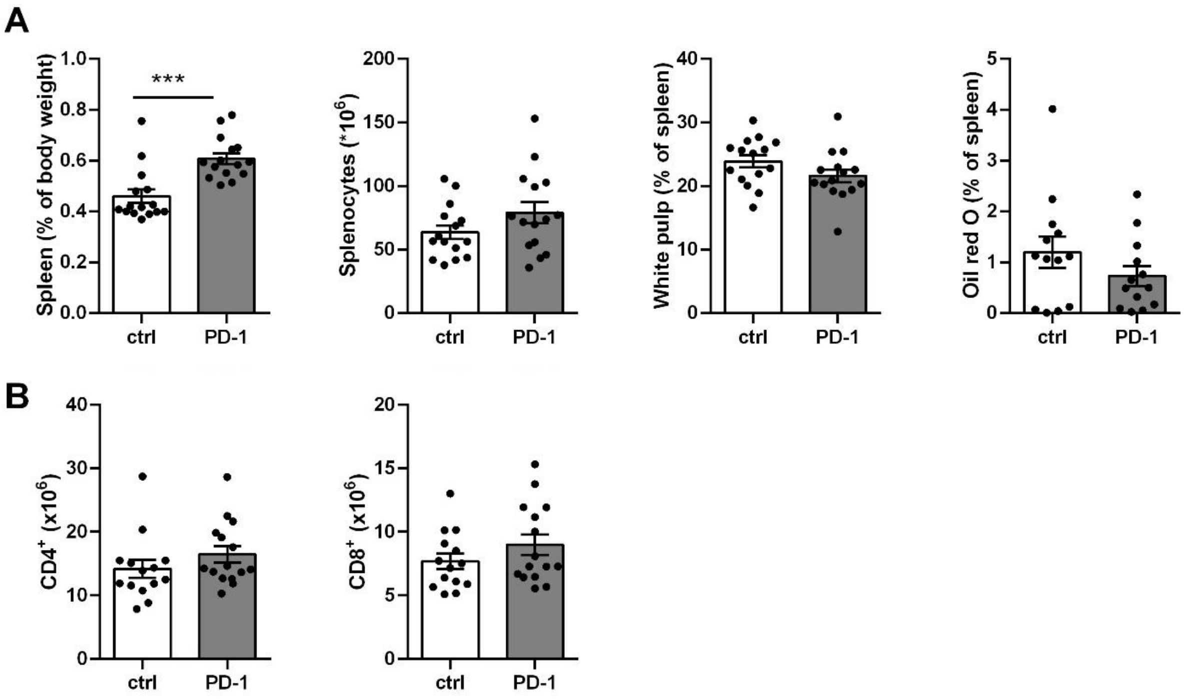

Figure S4: Enlarged spleen after 6 weeks of PD-1 stimulation, while similar splenocyte counts and white pulp content are found. Ldlr$^{-/-}$ mice were fed a WTD for 6 weeks while receiving a PD-1 agonist or control vehicle. The spleen as percentage of body weight is shown, as well as amount of isolated splenocytes, white pulp content and Oil-Red-O staining (A). Absolute values of CD4^{+} and CD8^{+} T cells did not differ between groups (B). Data are displayed as mean ± SEM. *** $p \leq 0.001$.

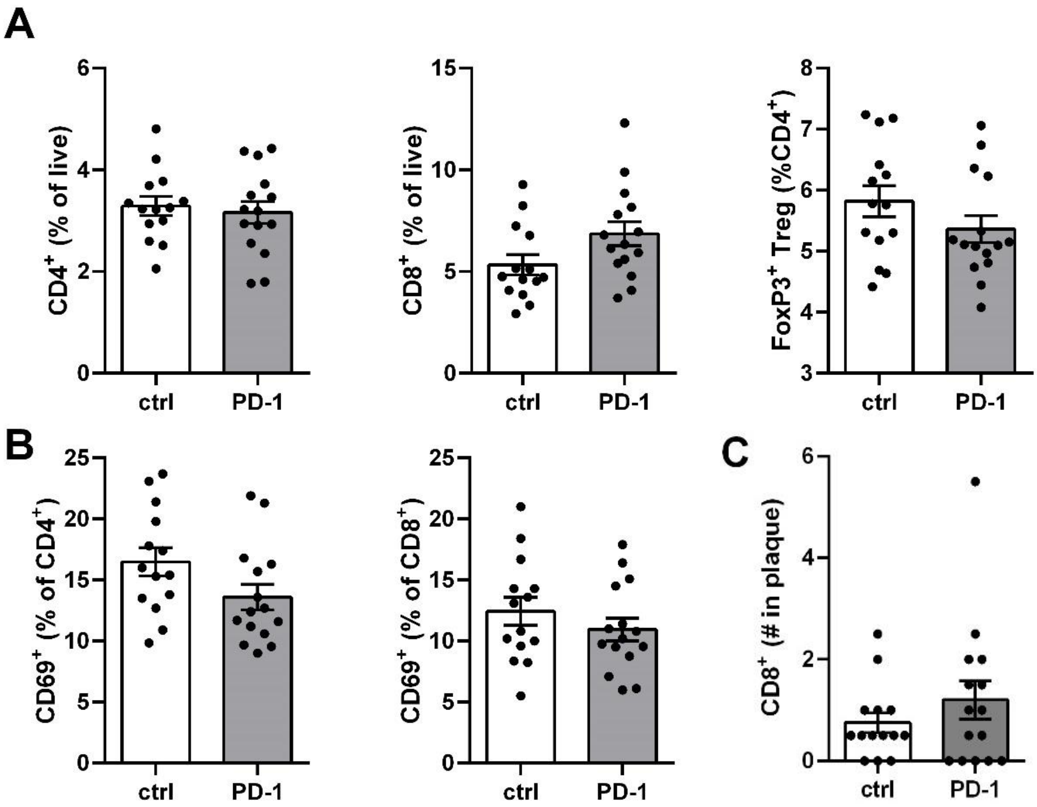

Figure S5: PD-1 stimulation for 6 weeks does not result in differences in circulating T cells Ldlr$^{-/-}$ mice were fed a WTD for 6 weeks while receiving a PD-1 agonist or control vehicle. Percentage of CD4^{+} and CD8^{+} T cells, and regulatory T cells in circulation, as assessed by flow cytometry (A). Expression of activation marker CD69 on circulating CD4^{+} and CD8^{+} T cells (B). CD8^{+} T cell numbers in the plaque did not differ between groups (C). Data are displayed as mean ± SEM.

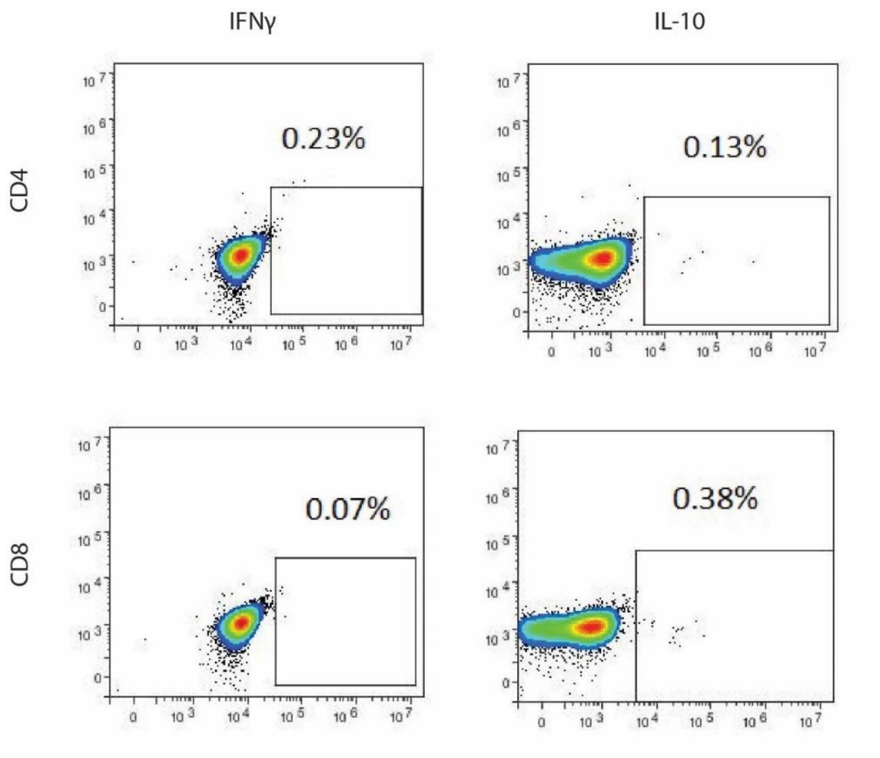

Figure S6: Flow cytometry controls for intracellular cytokine stainings in T cells. Ldlr$^{-/-}$ mice were fed a WTD for 6 weeks while receiving a PD-1 agonist or control vehicle. At sacrifice, splenocytes were isolated and stimulated with or without PMA/ionomycin/Bref A for 4 hours. Representative dot plots of IFNγ and IL-10 within unstimulated CD4^{+} and CD8^{+} T cells are shown as a control for the cytokine staining.
