# Supplementary figures and images for: Stimulation of the PD-1 Pathway Decreases Atherosclerotic Lesion Development in Ldlr Deficient Mice
Source: Front Cardiovasc Med. 2021 Nov 1;8:740531. doi: 10.3389/fcvm.2021.740531 (PMC8591266; doi:10.3389/fcvm.2021.740531)

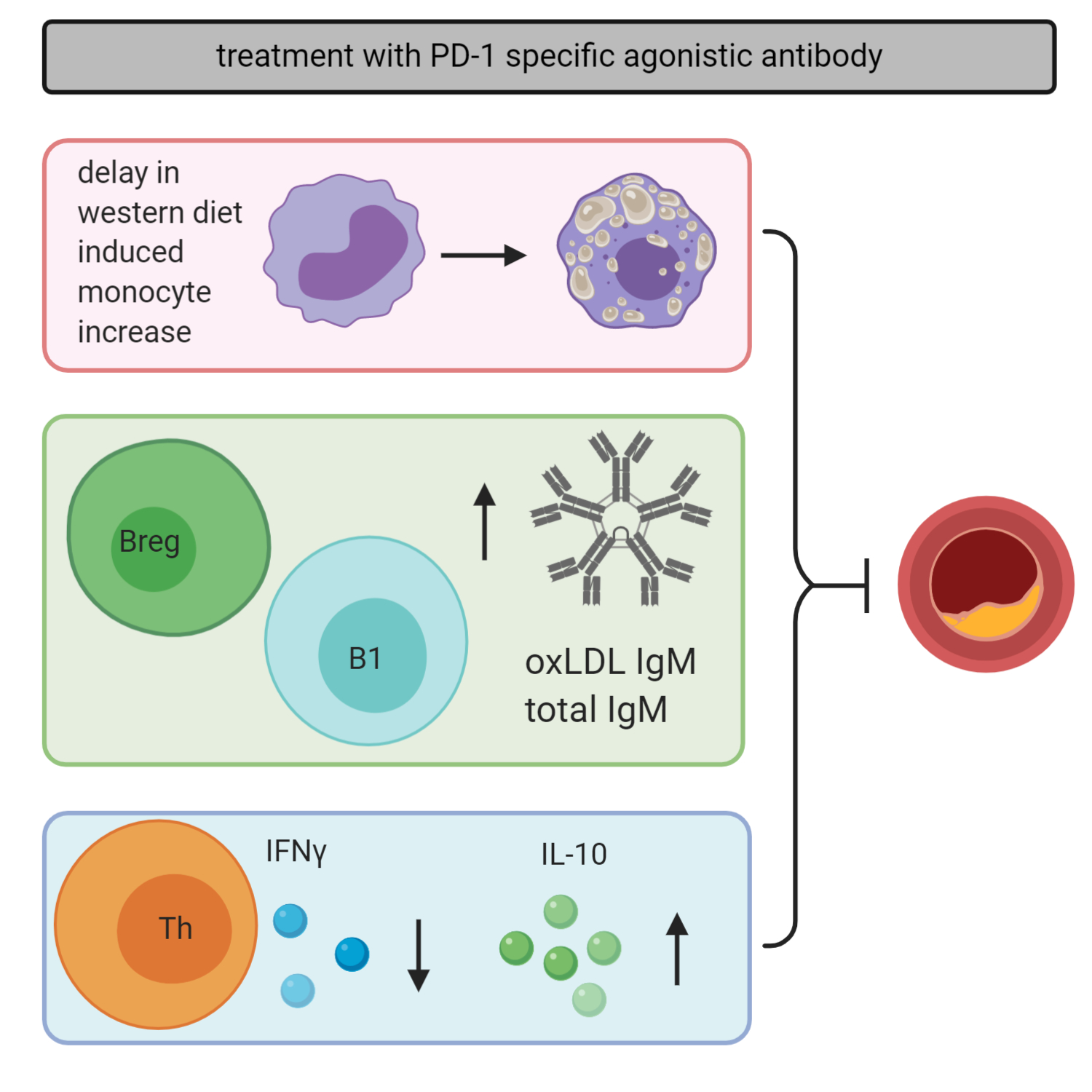

Supplement: Supplementary file 2 [file Image_1.JPEG]
